# Reinvigorating tumour-infiltrating lymphocytes from checkpoint inhibitor resistant melanomas

**DOI:** 10.1038/s41416-018-0218-3

**Published:** 2018-08-21

**Authors:** Inês Pires da Silva, Marcel Batten, Georgina V. Long

**Affiliations:** 1 Melanoma Institute Australia, Sydney, NSW Australia; 2 Sydney Medical School, The University of Sydney, Sydney, NSW Australia; 3 Royal North Shore Hospital, Sydney, NSW Australia

**Keywords:** Melanoma, Tumour immunology

## Summary

A recent trial of adoptive cell therapy for the treatment of patients who progressed on checkpoint inhibitors indicates that resistance is not a consequence of T cell ignorance. Tumour-reactive tumour-infiltrating lymphocytes (TILs) can be isolated from the majority of patients but tumour killing in vivo is short lived in most patients, reasserting the importance of the microenvironment in immunosuppression.

## Resistance to immunotherapy

In the past decade we have witnessed a change in the treatment of advanced melanoma, with targeted therapy (MAPK inhibitors) and immunotherapy (immune checkpoint inhibitors) significantly improving the overall survival of patients. Stimulating the immune system to induce cancer cell death, instead of using drugs that directly kill the cancer cells, has shown impressive long-term responses not only in melanoma, but also in other tumour types, including lung, kidney, head and neck, and Hodgkin’s Lymphoma. In advanced melanoma, the first generation of immune checkpoint inhibitors (CPI), anti-CTLA-4 (ipilimumab), induced long-term responses in 20% of the patients,^1^ whereas the second generation, anti-PD-1 (nivolumab or pembrolizumab), increased this number to approximately one-third of the patients.^2^ The highest response rate is achieved with the combination of anti-CTLA-4 and anti-PD-1 (response rate 58%);^3^ however, at least half of the patients have a melanoma that is resistant to this form of immunotherapy.^3^ An investigation by Andersen et al.^6^ has now provided new insights into the possibility of reactivating tumour-specific T cells from these CPI-resistant patients.

Several mechanisms of resistance to the dual immunotherapy combination have been described, and more than one of these mechanisms could coexist within the same patient. Although over-simplified, onco-immunologists have been dividing these resistant melanomas into ‘cold’ or ‘hot’, according to the absence or presence of tumour-infiltrating lymphocytes (TILs), respectively, to provide a mechanistic framework in which to study resistance. It is generally considered that a ‘cold’ tumour is not able to generate anti-tumour reactive T cells owing to a lack of neo-antigen expression, deficient antigen presentation (low MHC), or insufficient cytotoxic T lymphocytes (CTL) trafficking to the tumour. Alternately, a ‘hot’ but immunotherapy-resistant tumour contains anti-tumour reactive T cells, but they are not able to kill tumour cells. This is most likely as a consequence of suppressive mechanisms in the tumour microenvironment or functional inactivation intrinsic to the TILs due to the chronic antigenic stimulation. Immunosuppressive mechanisms that have been described frequently in the tumour microenvironment include the presence of immune suppressive cells (myeloid-derived suppressor cells, certain types of differentiated macrophages and regulatory T cells, which all act to inhibit cytotoxic T-cell responses), the secretion of immunosuppressive cytokines (IL-10 and TGFβ), the expression of other immune checkpoints (Tim-3, LAG3, ICOS) and a hostile metabolic environment (high IDO expression and hypoxia).^4^

## No intrinsic ignorance

Efforts to circumvent the problem of poor endogenous anti-tumour T-cell responses have included adoptive cell therapy (ACT). In this approach, TILs are isolated from resected tumour material, expanded and activated ex vivo, then returned to the patient. Although the variety of ACT trial structures has resulted in variable overall response rates, the potential is revealed by the complete responses frequently observed in a small percentage of patients.^5^ Andersen et al.^6^ have characterised the function of TILs from 23 advanced melanoma patients, who were resistant to prior anti-PD-1 (23/23 of the patients) and to anti-CTLA-4 (17/23 of the patients), in the context of an ACT clinical trial. Interestingly, et al. were able to isolate and expand TILs from all 23 patients, despite the poor response to previous CPI immunotherapy. After a conventional process of expansion, TILs (combined CD8 and CD4) were responsive to autologous melanoma cells in 19 out of 23 patients, which is consistent with previously reported data from CPI naïve patients.^7^

Success in isolating TILS from immunotherapy-refractory patients is also apparent in other cohorts.^8^ These data are of particular interest because they indicate that even in CPI-resistant patients, the T cells are not intrinsically ignorant to the tumour. It is important to point out that Andersen et al. observed a wide variation in the percentage of CD8 TILs that responded to tumour in vitro, with a relatively low median of 23%. This was even more pronounced for CD4 cells (4.5%). We may, therefore, need to focus attention on the phenotype and function of the remaining CD4 and CD8 T cells to define whether they have a pro-tumoural or anti-tumoural effect. Opportunity exists in studies such as this to more carefully define cell type and function after ex vivo stimulation and during the in vivo response.

**Fig. 1:**
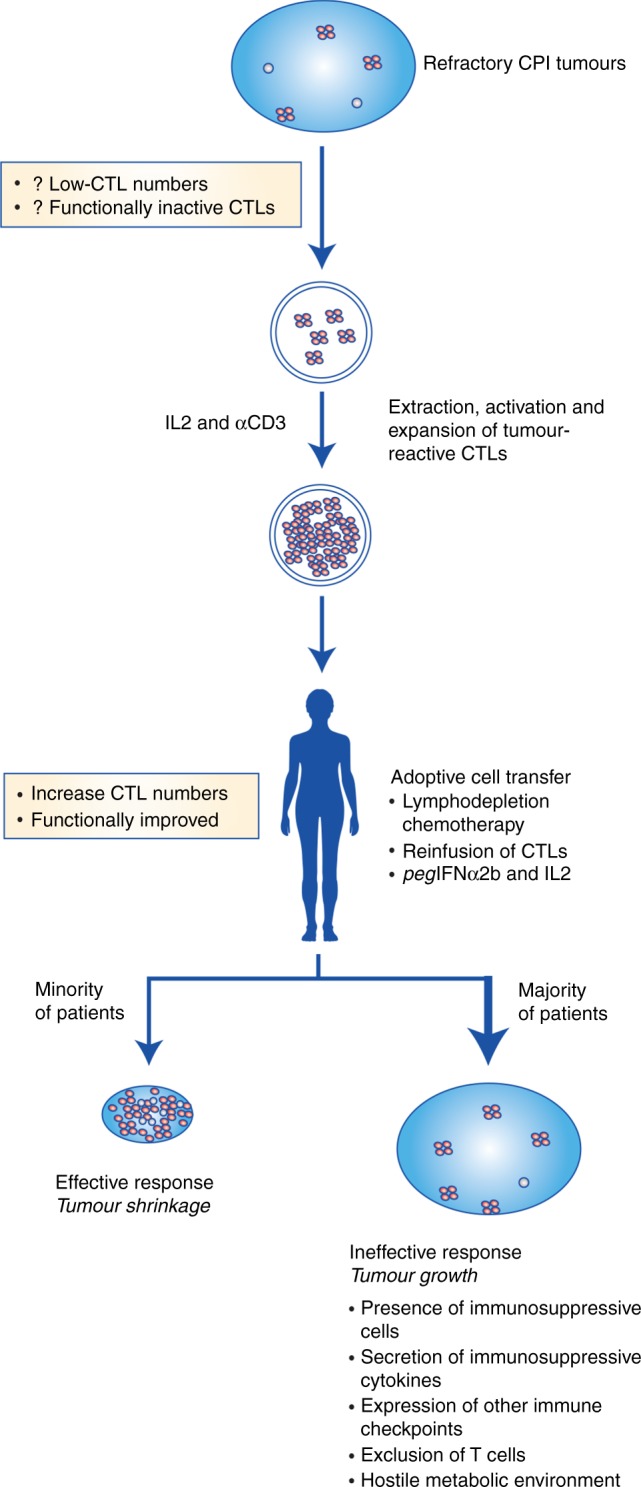
Extraction, activation and expansion of tumour-reactive cytotoxic T lymphocytes (CTLs). Anti-tumour reactive tumour-infiltrating lymphocytes (TILs) from checkpoint inhibitor (CPI) refractory patients can be isolated from excised tumours, activated and expanded. Reinfusion ensures the presence of numerous antigen-specific T cells in the body; however, they are not sufficiently effective in the majority of patients

When the TILs were re-infused (along with systemic IL-2 and *peg*IFNα2b), two out of twelve patients had an objective response. Importantly, the majority had some stability or shrinkage of the target lesions at the first clinical evaluation at 6 weeks. This confirms the in vivo anti-tumour activity of the approach, although the response was not durable in most cases. Thus, ex vivo stimulation is able to reinvigorate otherwise ineffective tumour-specific T cells in many immunotherapy-resistant patients (Fig. 1). The question that needs to be addressed now is why these initial responses do not last? Ex vivo expansion and activation of TILs effectively generates a ‘hot‘ tumour, so the answer probably lies with the above-mentioned mechanisms of resistance, including the presence of suppressive immune cells at the site, downregulation of MHC, the hostile metabolic environment and chronic antigen stimulation leading to ineffectual cytotoxic activity. In the case of the Andersen et al. study, supporting T cell proliferation and MHC expression through IL-2 and *peg*IFNα2b co-administration was not sufficient to maintain the response. Indeed, other processes such as tumour cell metabolism and the balance of dendritic cells and myeloid subpopulations in the tumour are defined modulators of ACT efficacy.^9,10^ Although we are able to increase the number of activated TILs with ACT, it is difficult to quantify how many of these traffic to the tumour microenvironment. It will also be important to understand how the TILs change during a failing anti-tumour response, with respect to cell persistence and functionality such as exhaustion or altered differentiation.

## Hope for future cancer therapy

Although this ACT phase I/II trial demonstrated a low response rate (16%), it was a small cohort of patients with multiple prior lines of treatment. More importantly, this study highlights the importance of the mechanisms of resistance in ‘hot’ tumours, and the urgent need for other therapeutic targets. The variety and the complexity of these described mechanisms suggest that each patient may have an individual pattern of resistance, which needs to be addressed with a more personalised treatment strategy. Effective combination drug therapies may come in a variety of forms and range from combining two immunotherapeutic strategies (for example ACT with an immune checkpoint inhibitor), or an immunotherapeutic with other strategies, including targeted therapy, chemotherapy or radiotherapy.

These recent findings are exciting because they show that TILs can be reinvigorated for clinical benefit from most patients, even those who are refractory to other immunotherapies. The challenge remains, to find ways to evade the immunosuppressive mechanisms innate to the tumour microenvironment.
